# Supplementary material for: The Genome-Wide Characterization of Alternative Splicing and RNA Editing in the Development of Coprinopsis cinerea
Source: J Fungi (Basel). 2023 Sep 9;9(9):915. doi: 10.3390/jof9090915 (PMC10532568; doi:10.3390/jof9090915)
Supplement: Supplementary file 1 [file jof-09-00915-s001.zip › Posttranscriptional_figuretable_v3.pdf]

Supplementary materials

Supplementary Datasets

Dataset S1. Gene count matrix by Trimmed Mean of M-values (TMM).
(DatasetS1_Gene_count_log2TMMmatrix.xlsx)

Dataset S2. Alternative splicing events and percent spliced in (PSI) value in *C. cinerea* development. (DatasetS2_Alternative_splicing.exp5.minor05.jad6.psi.xlsx)

Dataset S3. Alternatively spliced genes.
(DatasetS3_Alternative_splicing.exp5.minor05.jad6.feature.xlsx)

Dataset S4. RNA editing identified in *C. cinerea* development with matrix of editing level.
(DatasetS4_RNA_editing.editing_level.xlsx)

Dataset S5. Annotation of RNA editing sites found in *C. cinerea*.
(DatasetS5_RNA_editing.feature.xlsx)

Dataset S6. Full genome alignment of strain #326 genome (Muraguchi et al., 2015) to (Xie et al., 2021).
(DatasetS6_full_genome_alignment_of_Muraguchi2015_against_Xie2021.xlsx)

Supplementary Tables

Table S1. Sample collection, sequencing quality and alignment rate.
Table S2. Primers used in this study.
Table S3. Number of expressed genes in *C. cinerea* development, CPM > 1 in at least two replicates.
Table S4. Functional enrichment summary of DEGs.
Table S5. Wilcoxon test on PSI score of nine developmental stages.
Table S6. Number of RNA editing sites identified during *C. cinerea* development.
Table S7. Wilcoxon test on RNA editing levels of nine developmental stages.
Table S8. Wilcoxon test on RNA editing levels of twelve editing types.

Supplementary Figures

Figure S1. Cross dissection of pileus under high power microscope (400 ×).
Figure S2. Pie chart showing the summary of gene expression levels.
Figure S3. Volcano plot showing the distribution of gene expression changes.
Figure S4. KOG enrichment analysis on gene clusters.
Figure S5. KEGG enrichment analysis on gene clusters.
Figure S6. GO enrichment analysis on gene clusters.
Figure S7. PSI score of AS events identified in nine developmental stages.
Figure S8. PSI score of eight AS types in nine developmental stages.
Figure S9. PSI score of nine developmental stages in eight AS types.
Figure S10. Quantitative relation of gene expression and PSI value of alternatively spliced genes.
Figure S11. Scatter plots showing the changes on expression levels of alternative spliced genes and their PSI scores.
Figure S12. Summary on differentially spliced events between stages.
Figure S13. KOG annotation on developmentally regulated alternative splicing specific to the developmental process.
Figure S14. Common RNA editing sites among different stages.
Figure S15. Statistics on RNA editing sites identified from Muraguchi et al. (2015).
Figure S16. Common RNA editing sites identified from transcriptome data generated by Muraguchi et al. (2015).
Figure S17. Validation of RNA editing on hypothetical protein (CC2G_003350, scaffold_11: 1716350U>C).
Figure S18. Functional annotation of genes with RNA editing.
Figure S19. KO annotation of genes with RNA editing.
Figure S20. Expression levels of nucleoside deaminase annotated in *C. cinerea*.

Table S1. Sample collection, sequencing quality and alignment rate.

**Stage (Abb.)**	**Incubation**	**Sample**	**Clean reads**	**Clean bases**	**Q20 (%)**	**Q30 (%)**	**GC (%)**	**Mapping rate (%)**	**Mapped reads (deduplicated)**
**Vegetative mycelia (Myc)**	Continuous darkness at 37 °C for 4 d	Myc1	25,085,950	3,762,892,500	97.7	93.7	54.5	93.87	15,936,386
		Myc2	32,513,954	4,877,093,100	97.8	93.9	54.5	94.28	20,024,836
		Myc3	26,208,640	3,931,296,000	97.7	93.7	54.5	94.38	17,280,727
**Oidia forming mycelia (Oidia)**	Continuous light at 37 °C for 4 d	Oidia1	31,417,198	4,712,579,700	97.5	93.2	54.1	95.01	20,476,670
		Oidia2	30,190,208	4,528,531,200	97.8	93.8	54.3	95.06	19,248,283
		Oidia3	29,196,406	4,379,460,900	97.7	93.7	54.3	94.58	18,912,548
**Sclerotia forming mycelia (Scl)**	Continuous darkness at 37 °C for 12 d	Scl1	32,972,822	4,945,923,300	94.1	85.7	53.9	92.55	21,480,526
		Scl2	37,357,474	5,603,621,100	95.0	87.6	54.0	92.89	24,696,908
		Scl3	29,848,036	4,477,205,400	94.4	86.1	54.0	93.14	20,434,835
**Mycelia with hyphal knots (Knot)**	Continuous darkness at 37 °C for 5.5 d, and 12 h:12 h light–dark cycle for 1 d	Knot1	28,412,998	4,261,949,700	97.8	93.8	54.0	94.43	18,137,097
		Knot2	31,571,766	4,735,764,900	97.7	93.7	54.3	93.65	18,237,961
		Knot3	34,713,178	5,206,976,700	97.8	94.0	54.5	93.39	20,334,292
**Primordia undergoing meiosis (Pri)**	Continuous darkness at 37 °C for 5.5 d, and 12 h:12 h light–dark cycle for 6 d	Pri1	24,910,904	3,736,635,600	97.8	93.9	54.0	94.78	16,965,939
		Pri2	24,551,978	3,682,796,700	97.7	93.8	54.2	94.26	17,247,702
		Pri3	25,494,022	3,824,103,300	97.4	92.8	54.2	94.26	18,233,166
**Young fruiting bodies undergoing spore formation (YFB)**	Continuous darkness at 37 °C for 5.5 d, and 12 h:12 h light–dark cycle for 6.5 d	YFB1	26,599,914	3,989,987,100	97.7	93.7	54.1	94.92	17,311,918
		YFB2	24,264,552	3,639,682,800	97.7	93.8	54.2	94.56	16,574,052
		YFB3	28,400,844	4,260,126,600	97.8	93.8	54.1	95.31	18,221,676
**Mature basidiospores (BS)**	Basidiospore discharged from mature cap	BS1	17,064,684	2,546,579,938	96.6	91.1	54.2	93.60	12,093,628
		BS2	17,492,502	2,606,856,924	96.6	90.9	54.0	93.46	10,770,048
		BS3	17,355,936	2,586,947,642	96.7	91.1	54.2	93.78	11,111,413
**Half germinating basidiospores (BS12h)**	Continuous darkness at 37 °C for 12 h, broth 150 rpm	BS12h1	17,126,036	2,546,749,128	94.3	87.6	45.9	9.28	832,554
		BS12h2	17,158,542	2,552,883,760	95.2	88.9	49.3	27.17	2,431,668
		BS12h3	17,244,842	2,571,283,772	95.6	89.8	49.5	29.61	2,544,746
**Fully germinated basidiospores (BS24h)**	Continuous darkness at 37 °C for 24 h, broth 150 rpm	BS24h1	17,162,606	2,556,983,164	96.6	91.0	54.5	93.12	11,181,138
		BS24h2	16,525,520	2,461,013,426	96.7	91.3	54.5	93.08	10,628,646
		BS24h3	16,917,964	2,519,857,620	95.9	90.0	52.0	61.54	6,686,954
**Genomic DNA**	Continuous darkness at 37 °C	DNA	9,213,544	1,382,031,600	98.0	94.4	50.3	98.84	8,068,437

Table S2. Primers used in this study.

	Target		Sequence (5’ →3’)
**PCR-Sanger**	Hypothetical protein	F	TTCAGCGTCTTTCGCTGCAAT
	(CC2G_003350, scaffold_11: 1716350T>C)	R	CTCATCGAACGCAAGTTTCGG
	Spliceosomal U1 snRNP C	F	ACTCACGACTCGGCTTCAG
	(CC2G_010208)	R	TGGGCCGTTAAATCCACCTG
	STE/STE11 protein kinase	F	GGTGGACTATCTGCCTGGTC
	(CC2G_011916)	R	GCCACTTTCCCTTACGGAGG
	RhoGAP	F	CTGGAGCTAGCCAACCTTCG
	(CC2G_009456)	R	CAACACAGCACGGGTTTGAC
**qRT-PCR**	18S rRNA	F	GCCTGTTTGAGTGTCATTAAATTCTC
		R	CTGCAACCCCCACATCCA
	Adenosine deaminase-like protein	F	GAGAGTGCCTGGACATAGCC
	(CC2G_001628)	R	CGCAAAGAACGTCTGGAACG
	Guanine deaminase	F	ACCACGACAACCTTCTACGG
	(CC2G_005289)	R	TCGATGTTTCCGGTTGGTCC
	adenosine deaminase	F	GGTCAACGCCCAGGTTCTTA
	(CC2G_005434)	R	ATACTCGTCGCTGCATGTCC
	tRNA specific adenosine deaminase	F	ATCGATTGCATCCTCAGCGA
	(CC2G_010905)	R	TGATGCACGGCTCTACTGTG
	cytosine deaminase-uracil phosphoribosyltransferase deaminase	F	AAGTGCTCATCGAACACGGT
	(CC2G_011103)	R	TCACTTTGAGCGACGGGAAG
	cytidine deaminase	F	CGTATTCGCGGTTTCCTGTG
	(CC2G_012163)	R	CAGCGCAGATAGTTCCACCA
	AMP deaminase	F	GTACAGCCTGCTGAACGAGT
	(CC2G_012607)	R	TGGACTTGATGAACCGGAGC
	tRNA specific adenosine deaminase	F	TGCTTTGGGACTCGGTTCTC
	(CC2G_012760)	R	TTCTCCAGGTTCGATGTCGC

Table S3. Number of expressed genes in *C. cinerea* development, CPM > 1 in at least two replicates.

**Stage**	**(Abb.)**	**Number of genes**
Vegetative mycelia	(Myc)	11,333
Oidia forming mycelia	(Oidia)	11,269
Sclerotia forming mycelia	(Scl)	11,075
Mycelia with hyphal knots	(Knot)	11,396
Primordia undergoing meiosis	(Pri)	11,392
Young fruiting bodies undergoing spore formation	(YFB)	10,722
Mature basidiospores	(BS)	10,043
Half germinating basidiospores	(BS12h)	10,301
Fully germinated basidiospores	(BS24h)	10,060
Total		12,450

Table S4. Functional enrichment summary of DEGs.

Groups	ID	Description	ONTOLOGY
cluster1	GO:0020037	heme binding	Molecular Function
cluster1	GO:0004553	hydrolase activity, hydrolyzing O-glycosyl compounds	Molecular Function
cluster2	GO:0020037	heme binding	Molecular Function
cluster2	GO:0030248	cellulose binding	Molecular Function
cluster2	GO:0016831	carboxy-lyase activity	Molecular Function
cluster2	GO:0046872	metal ion binding	Molecular Function
cluster5	GO:0051539	4 iron, 4 sulfur cluster binding	Molecular Function
cluster5	GO:0008168	methyltransferase activity	Molecular Function
cluster6	GO:0003723	RNA binding	Molecular Function
cluster1	GO:0016021	integral component of membrane	Cellular Component
cluster2	GO:0016021	integral component of membrane	Cellular Component
cluster2	GO:0005618	cell wall	Cellular Component
cluster2	GO:0009277	fungal-type cell wall	Cellular Component
cluster3	GO:0016021	integral component of membrane	Cellular Component
cluster3	GO:0098803	respiratory chain complex	Cellular Component
cluster3	GO:0098800	inner mitochondrial membrane protein complex	Cellular Component
cluster3	GO:0000323	lytic vacuole	Cellular Component
cluster4	GO:0016021	integral component of membrane	Cellular Component
cluster4	GO:1905369	endopeptidase complex	Cellular Component
cluster4	GO:0005839	proteasome core complex	Cellular Component
cluster5	GO:0005730	nucleolus	Cellular Component
cluster5	GO:0043232	intracellular non-membrane-bounded organelle	Cellular Component
cluster5	GO:0005840	ribosome	Cellular Component
cluster5	GO:0043231	intracellular membrane-bounded organelle	Cellular Component
cluster5	GO:0070013	intracellular organelle lumen	Cellular Component
cluster6	GO:0005840	ribosome	Cellular Component
cluster6	GO:0043232	intracellular non-membrane-bounded organelle	Cellular Component
cluster6	GO:0005739	mitochondrion	Cellular Component

Table S4. Functional enrichment summary of DEGs (continued).

Groups	ID	Description	ONTOLOGY
cluster3	GO:0043436	oxoacid metabolic process	Biological Process
cluster3	GO:1901605	alpha-amino acid metabolic process	Biological Process
cluster3	GO:0055086	nucleobase-containing small molecule metabolic process	Biological Process
cluster3	GO:0006520	cellular amino acid metabolic process	Biological Process
cluster3	GO:0072524	pyridine-containing compound metabolic process	Biological Process
cluster3	GO:0006753	nucleoside phosphate metabolic process	Biological Process
cluster4	GO:0007186	G protein-coupled receptor signaling pathway	Biological Process
cluster5	GO:0022613	ribonucleoprotein complex biogenesis	Biological Process
cluster5	GO:1901566	organonitrogen compound biosynthetic process	Biological Process
cluster5	GO:0016053	organic acid biosynthetic process	Biological Process
cluster6	GO:0006518	peptide metabolic process	Biological Process
cluster6	GO:0043603	cellular amide metabolic process	Biological Process
cluster6	GO:1901566	organonitrogen compound biosynthetic process	Biological Process
cluster6	GO:0044267	cellular protein metabolic process	Biological Process
cluster6	GO:0010467	gene expression	Biological Process
cluster6	GO:0019538	protein metabolic process	Biological Process

Table S5. Wilcoxon test on PSI score of nine developmental stages.

Group1	Group2	p	p.adj	p.format	p.signif
BS	BS12h	0.858611	1	0.85861	ns
BS	Oidia	8.24E-07	2.90E-05	8.20E-07	****
BS	Scl	4.15E-06	0.00014	4.10E-06	****
BS	BS12h	3.18E-07	1.10E-05	3.20E-07	****
BS	Knot	6.55E-06	0.00022	6.60E-06	****
BS	Myc	0.000219	0.0068	0.00022	***
BS	YFB	0.002648	0.079	0.00265	**
BS	Pri	0.01848	0.5	0.01848	*
BS	BS24h	1.86E-05	6.00E-04	1.90E-05	****
Oidia	Scl	0.723552	1	0.72355	ns
Oidia	BS12h	0.867384	1	0.86738	ns
Oidia	Knot	0.578461	1	0.57846	ns
Oidia	Myc	0.253901	1	0.2539	ns
Oidia	YFB	0.03807	0.89	0.03807	*
Oidia	Pri	0.011542	0.32	0.01154	*
Oidia	BS24h	0.850476	1	0.85048	ns
Scl	BS12h	0.642752	1	0.64275	ns
Scl	Knot	0.854224	1	0.85422	ns
Scl	Myc	0.439598	1	0.4396	ns
Scl	YFB	0.085381	1	0.08538	ns
Scl	Pri	0.028827	0.72	0.02883	*
Scl	BS24h	0.891506	1	0.89151	ns
BS12h	Knot	0.466307	1	0.46631	ns
BS12h	Myc	0.207701	1	0.2077	ns
BS12h	YFB	0.025464	0.66	0.02546	*
BS12h	Pri	0.007177	0.21	0.00718	**
BS12h	BS24h	0.757683	1	0.75768	ns
Knot	Myc	0.524763	1	0.52476	ns
Knot	YFB	0.12103	1	0.12103	ns
Knot	Pri	0.040944	0.9	0.04094	*
Knot	BS24h	0.759005	1	0.759	ns
Myc	YFB	0.387848	1	0.38785	ns
Myc	Pri	0.179955	1	0.17996	ns
Myc	BS24h	0.393579	1	0.39358	ns
YFB	Pri	0.591205	1	0.59121	ns
YFB	BS24h	0.092002	1	0.092	ns
Pri	BS24h	0.036983	0.89	0.03698	*

Table S6. Number of RNA editing sites identified during *C. cinerea* development.

**Stage**	**RE sites**	**Stage-specific RE sites**	**Mean editing level**	**Sample**	**RE events**
BS	24	13	0.143	BS1	13
				BS2	18
				BS3	19
BS12h	7	4	0.0879	BS12h1	2
				BS12h2	6
				BS12h3	6
BS24h	17	4	0.0685	BS24h1	17
				BS24h2	17
				BS24h3	0
Myc	29	12	0.100	Myc1	22
				Myc2	23
				Myc3	19
Oidia	24	6	0.105	Oidia1	18
				Oidia2	19
				Oidia3	17
Scl	141	97	0.0665	Scl1	114
				Scl2	119
				Scl3	100
Knot	33	13	0.0685	Knot1	23
				Knot2	31
				Knot3	23
Pri	21	3	0.0872	Pri1	15
				Pri2	17
				Pri3	18
YFB	22	11	0.0849	YFB1	20
				YFB2	14
				YFB3	15
Total	217	163	0.0810		726

Table S7. Wilcoxon test on RNA editing levels of nine developmental stages.

Group1	Group2	p	p.adj	p.format	p.signif
Pri	Scl	0.65	1	0.65	ns
Pri	Oidia	0.76	1	0.76	ns
Pri	YFB	0.77	1	0.77	ns
Pri	BS	0.10	1	0.10	ns
Pri	BS24h	0.12	1	0.12	ns
Pri	Knot	0.56	1	0.56	ns
Pri	Myc	0.52	1	0.52	ns
Pri	BS12h	0.04	1	0.04	*
Scl	Oidia	0.92	1	0.92	ns
Scl	YFB	0.37	1	0.37	ns
Scl	BS	0.07	1	0.07	ns
Scl	BS24h	0.11	1	0.11	ns
Scl	Knot	0.70	1	0.70	ns
Scl	Myc	0.67	1	0.67	ns
Scl	BS12h	0.04	1	0.04	*
Oidia	YFB	0.56	1	0.56	ns
Oidia	BS	0.18	1	0.18	ns
Oidia	BS24h	0.20	1	0.20	ns
Oidia	Knot	0.70	1	0.70	ns
Oidia	Myc	0.76	1	0.76	ns
Oidia	BS12h	0.08	1	0.08	ns
YFB	BS	0.05	1	0.05	ns
YFB	BS24h	0.06	1	0.06	ns
YFB	Knot	0.32	1	0.32	ns
YFB	Myc	0.33	1	0.33	ns
YFB	BS12h	0.02	0.81	0.02	*
BS	BS24h	0.97	1	0.97	ns
BS	Knot	0.21	1	0.22	ns
BS	Myc	0.27	1	0.27	ns
BS	BS12h	0.40	1	0.40	ns
BS24h	Knot	0.24	1	0.24	ns
BS24h	Myc	0.34	1	0.34	ns
BS24h	BS12h	0.34	1	0.34	ns
Knot	Myc	0.93	1	0.93	ns
Knot	BS12h	0.07	1	0.07	ns
Myc	BS12h	0.12	1	0.12	ns

Table S8. Wilcoxon test on RNA editing levels of twelve editing types.

Group 1	Group 2	P	P adj	P format	P signif	Group 1	Group 2	P	P adj	P format	P signif
TC	AG	0.24	1	0.24	ns	CT	TG	0.85	1	0.85	ns
TC	CG	0.02	1	0.02	*	CT	AC	0.05	1	0.05	ns
TC	CT	0.47	1	0.47	ns	CT	GC	0.07	1	0.07	ns
TC	GA	0.29	1	0.29	ns	CT	TA	0.93	1	0.93	ns
TC	GT	0.05	1	0.05	ns	CT	CA	0.72	1	0.72	ns
TC	AT	0.01	0.33	0.01	**	GA	GT	0.14	1	0.14	ns
TC	TG	0.87	1	0.87	ns	GA	AT	0.01	0.5	0.01	**
TC	AC	0.06	1	0.06	ns	GA	TG	0.84	1	0.84	ns
TC	GC	0.03	1	0.03	*	GA	AC	0.15	1	0.15	ns
TC	TA	0.82	1	0.82	ns	GA	GC	0.01	0.5	0.01	**
TC	CA	0.96	1	0.96	ns	GA	TA	0.68	1	0.68	ns
AG	CG	0.03	1	0.03	*	GA	CA	0.75	1	0.75	ns
AG	CT	0.09	1	0.09	ns	GT	AT	0.15	1	0.15	ns
AG	GA	0.92	1	0.92	ns	GT	TG	0.39	1	0.39	ns
AG	GT	0.13	1	0.13	ns	GT	AC	0.65	1	0.65	ns
AG	AT	0.00	0.24	0.00	**	GT	GC	0.01	0.41	0.01	**
AG	TG	0.78	1	0.78	ns	GT	TA	0.32	1	0.32	ns
AG	AC	0.07	1	0.07	ns	GT	CA	0.24	1	0.24	ns
AG	GC	0.01	0.42	0.01	**	AT	TG	0.07	1	0.07	ns
AG	TA	0.69	1	0.69	ns	AT	AC	0.65	1	0.65	ns
AG	CA	0.69	1	0.69	ns	AT	GC	0.00	0.2	0.00	**
CG	CT	0.01	0.46	0.01	**	AT	TA	0.07	1	0.07	ns
CG	GA	0.04	1	0.04	*	AT	CA	0.03	1	0.03	*
CG	GT	0.52	1	0.52	ns	TG	AC	0.17	1	0.17	ns
CG	AT	0.33	1	0.33	ns	TG	GC	0.64	1	0.64	ns
CG	TG	0.23	1	0.23	ns	TG	TA	0.70	1	0.70	ns
CG	AC	0.84	1	0.84	ns	TG	CA	1.00	1	1.00	ns
CG	GC	0.01	0.38	0.01	**	AC	GC	0.02	1	0.02	*
CG	TA	0.21	1	0.21	ns	AC	TA	0.19	1	0.19	ns
CG	CA	0.10	1	0.10	ns	AC	CA	0.17	1	0.17	ns
CT	GA	0.13	1	0.13	ns	GC	TA	0.53	1	0.53	ns
CT	GT	0.02	1	0.02	*	GC	CA	0.22	1	0.22	ns
CT	AT	0.00	0.16	0.00	**	TA	CA	1.00	1	1.00	ns

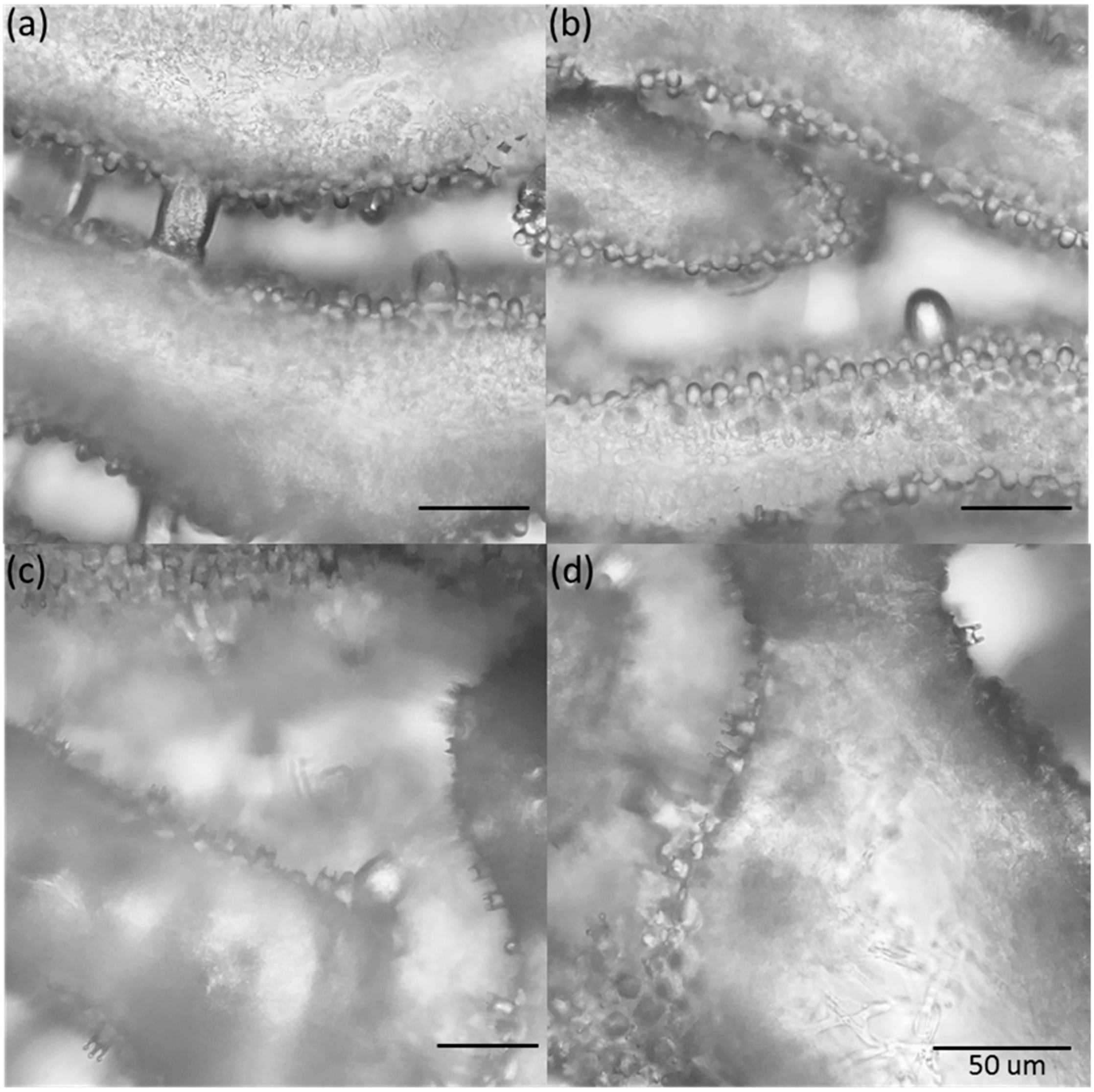

Figure S1. Cross dissection of pileus under high power microscope (400 ×). (a) and (b) Primordium contains the meiosis tissue. (c) and (d) Young fruiting body contains the tissue undergoing basidiospore formation.

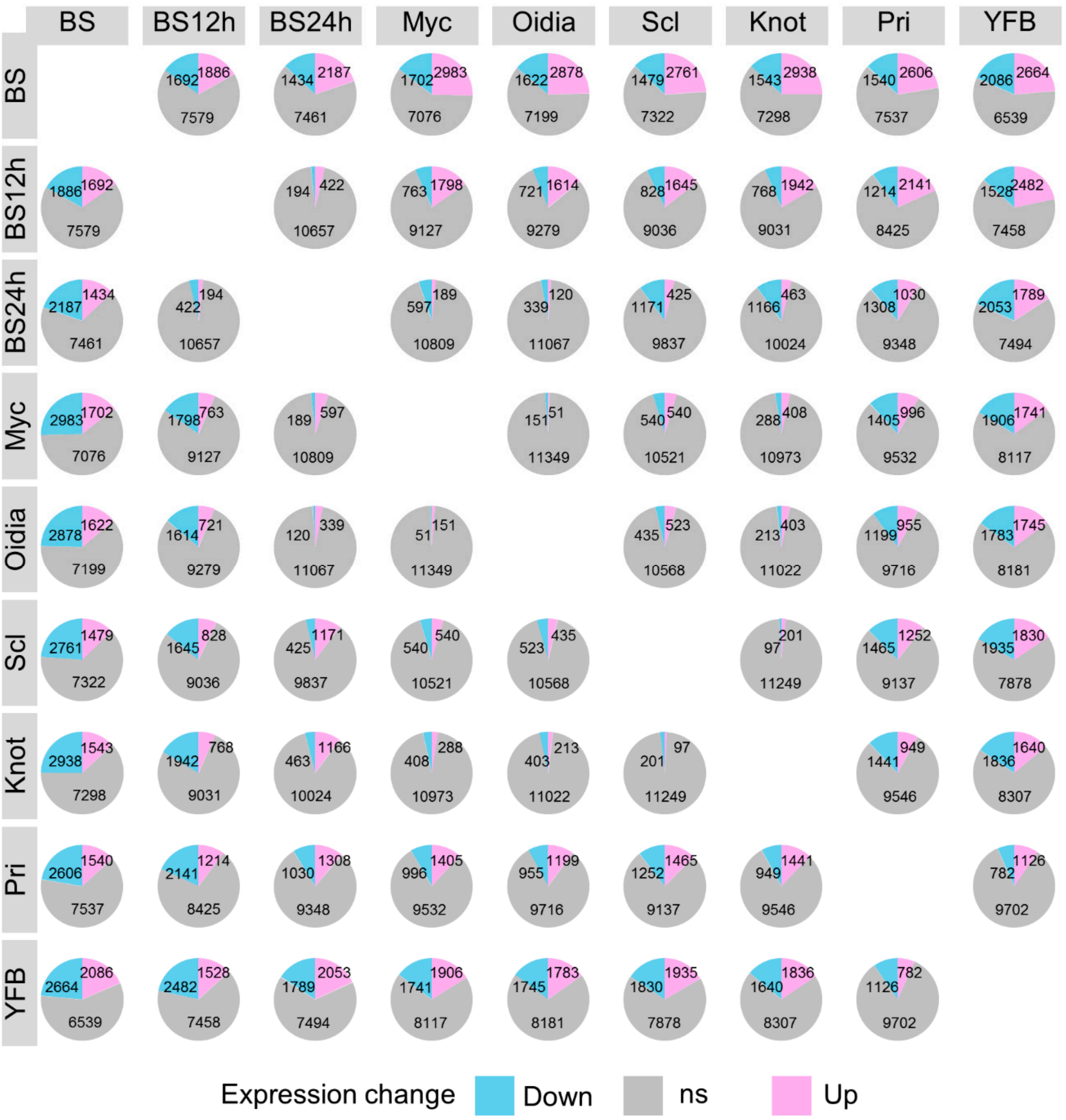

Figure S2. Pie chart showing the summary of gene expression levels. Sample of each column were compared against sample of each row, for example, compare to BS, 1,886 genes were up-regulated, and 1,692 genes were down-regulated in BS12h.

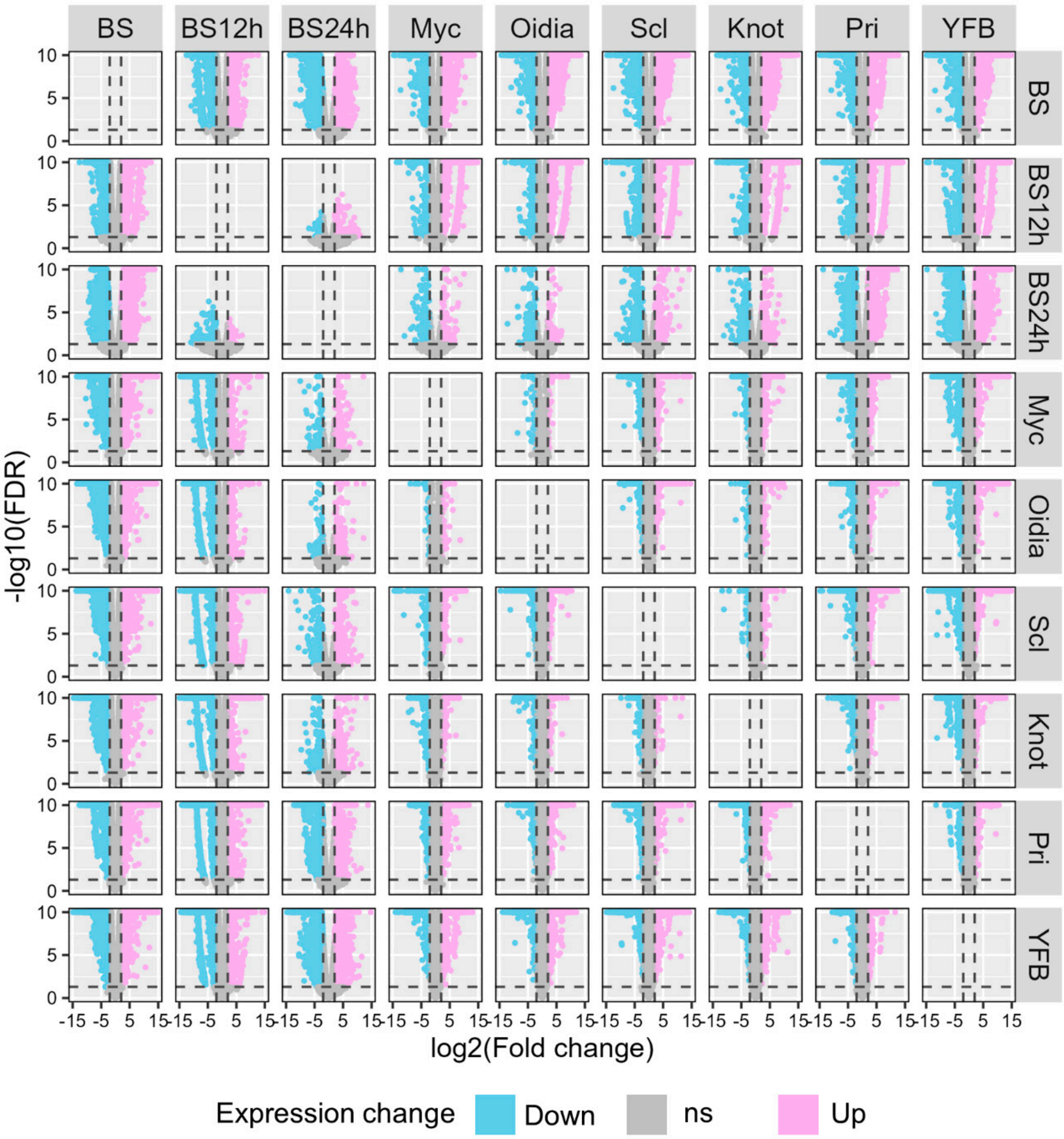

Figure S3. Volcano plot showing the distribution of gene expression changes. Sample of each column were compared against sample of each row, for example, compare to BS, 1,886 genes were up-regulated, and 1,692 genes were down-regulated in BS12h.

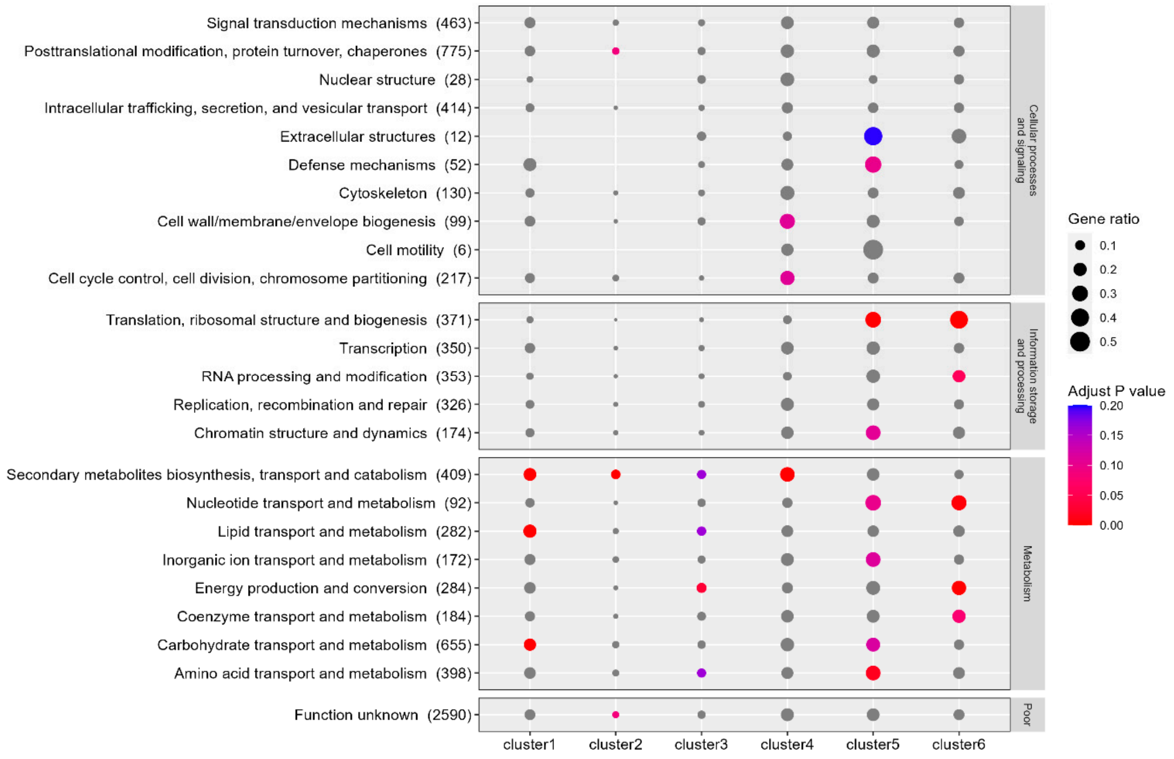

Figure S4. KOG enrichment analysis on gene clusters. Numbers of genes annotated to specific KOG term are listed beside the term. Gene ratio is calculated by annotated genes of specific KOG term in each cluster over annotated genes of specific KOG term in the genome background. Enriched groups with Benjamini and Hochberg method (BH) adjusted p value ≤ 0.20 are coloured red to blue, others are in grey.

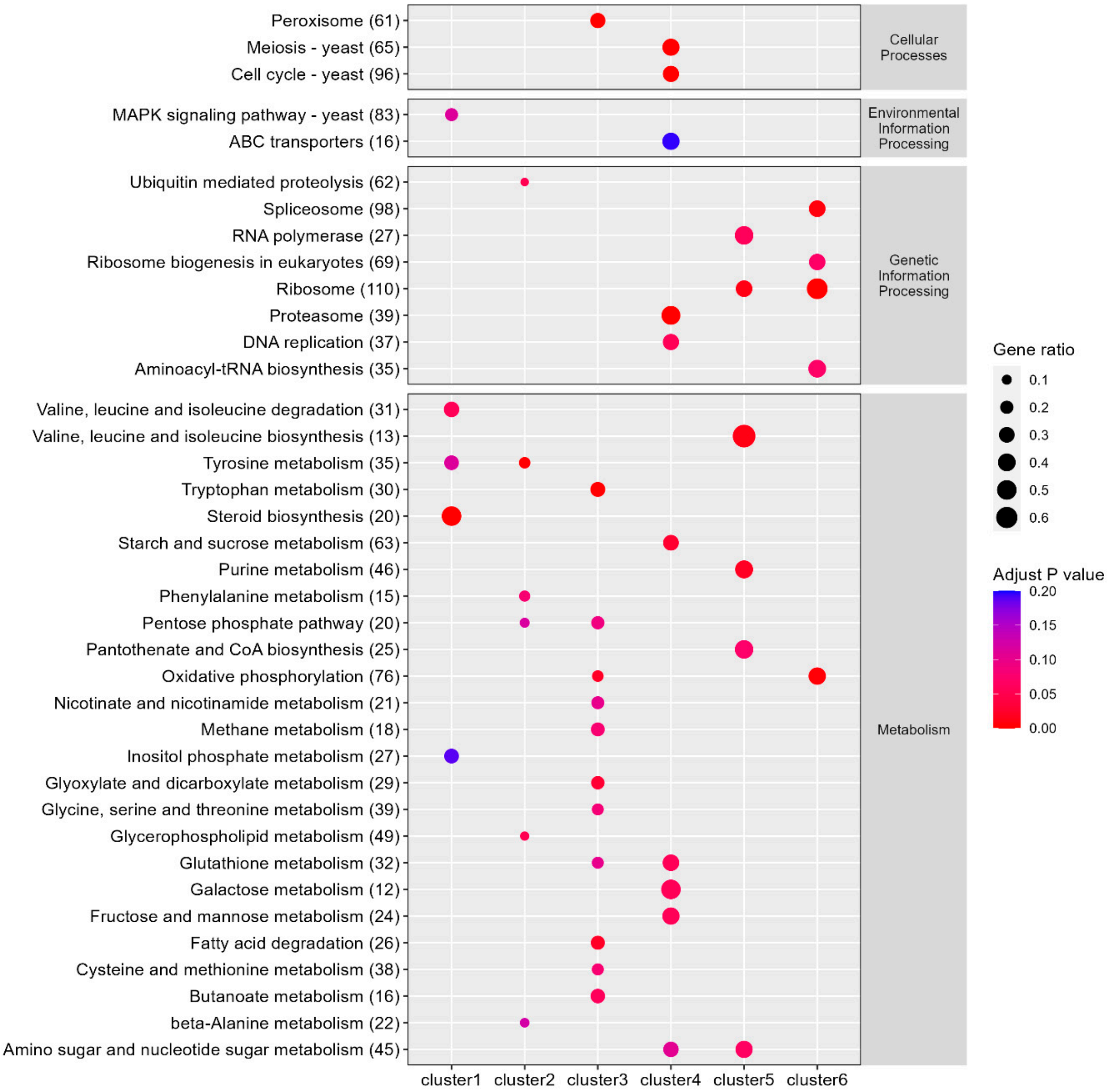

Figure S5. KEGG enrichment analysis on gene clusters. Numbers of genes annotated to specific KEGG pathway are listed beside the term. Gene ratio is calculated by annotated genes of specific KEGG pathway in each cluster over annotated genes of specific KEGG pathway in the genome background. Enriched groups with Benjamini and Hochberg method (BH) adjusted p value ≤ 0.20 are coloured red to blue.

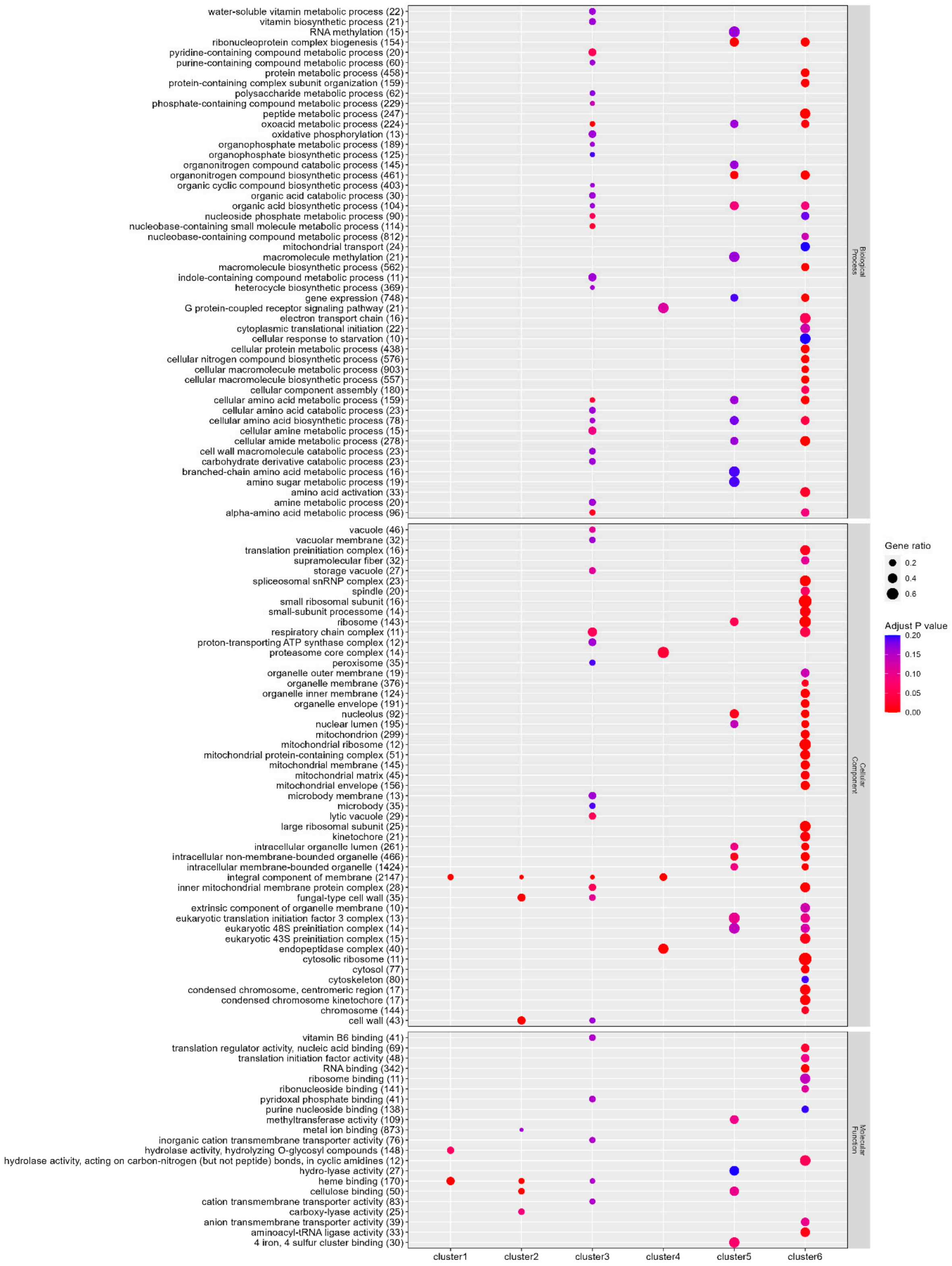

Figure S6. GO enrichment analysis on gene clusters. Numbers of genes annotated to specific GO term are listed beside the term. Gene ratio is calculated by annotated genes of specific GO term in each cluster over annotated genes of specific GO term in the genome background. Terms are filtered to GO level 5. Enriched groups with Benjamini and Hochberg method (BH) adjusted p value ≤ 0.20 are coloured red to blue.

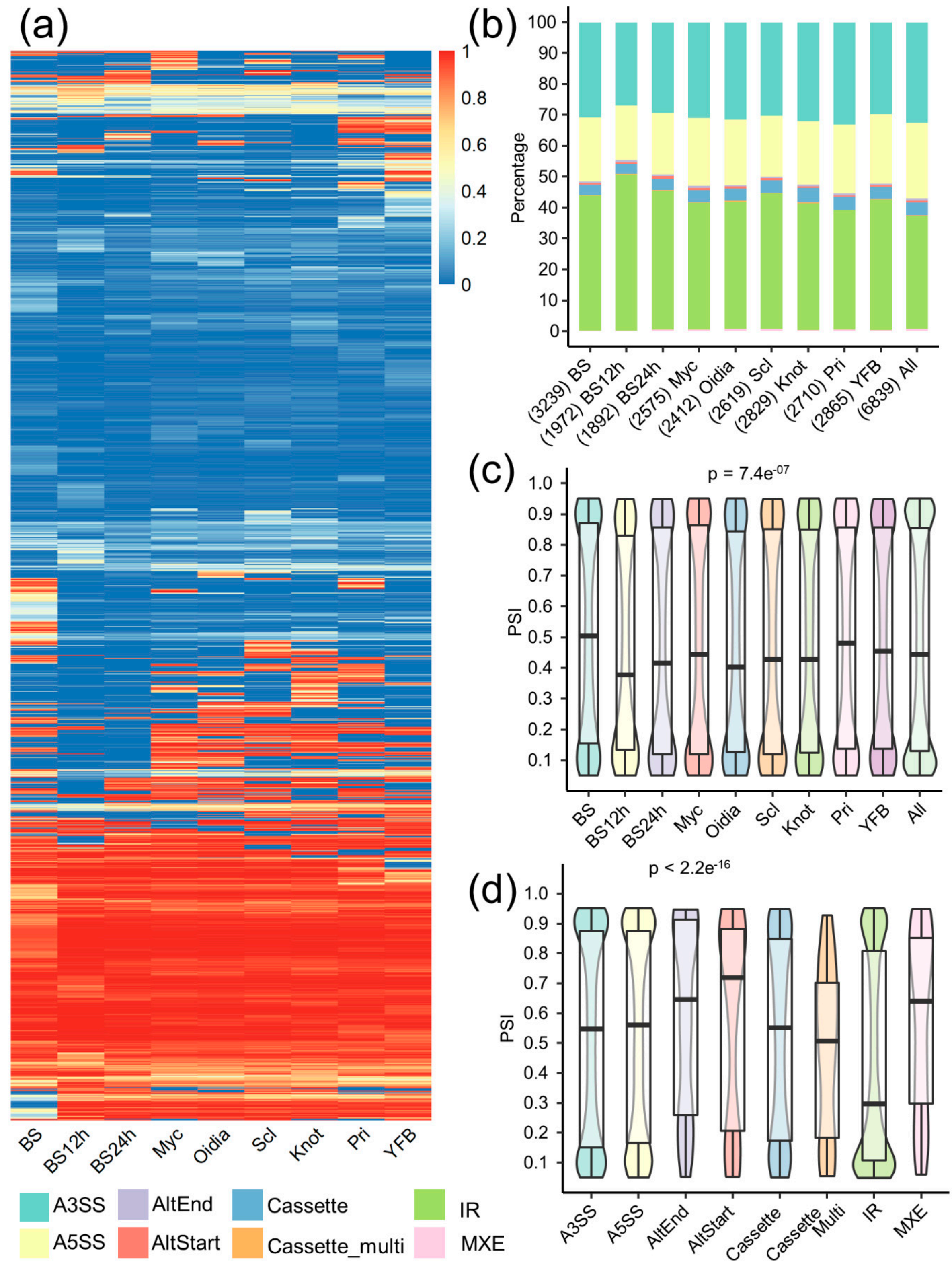

Figure S7. PSI score of AS events identified in nine developmental stages. (a) Heatmap showing the PSI score; (b) Distribution of eight AS types in nine stages; (c) PSI score of different stages, statistical results were detailed in table S5; (d) Violin plot summarising the PSI score of each AS types.

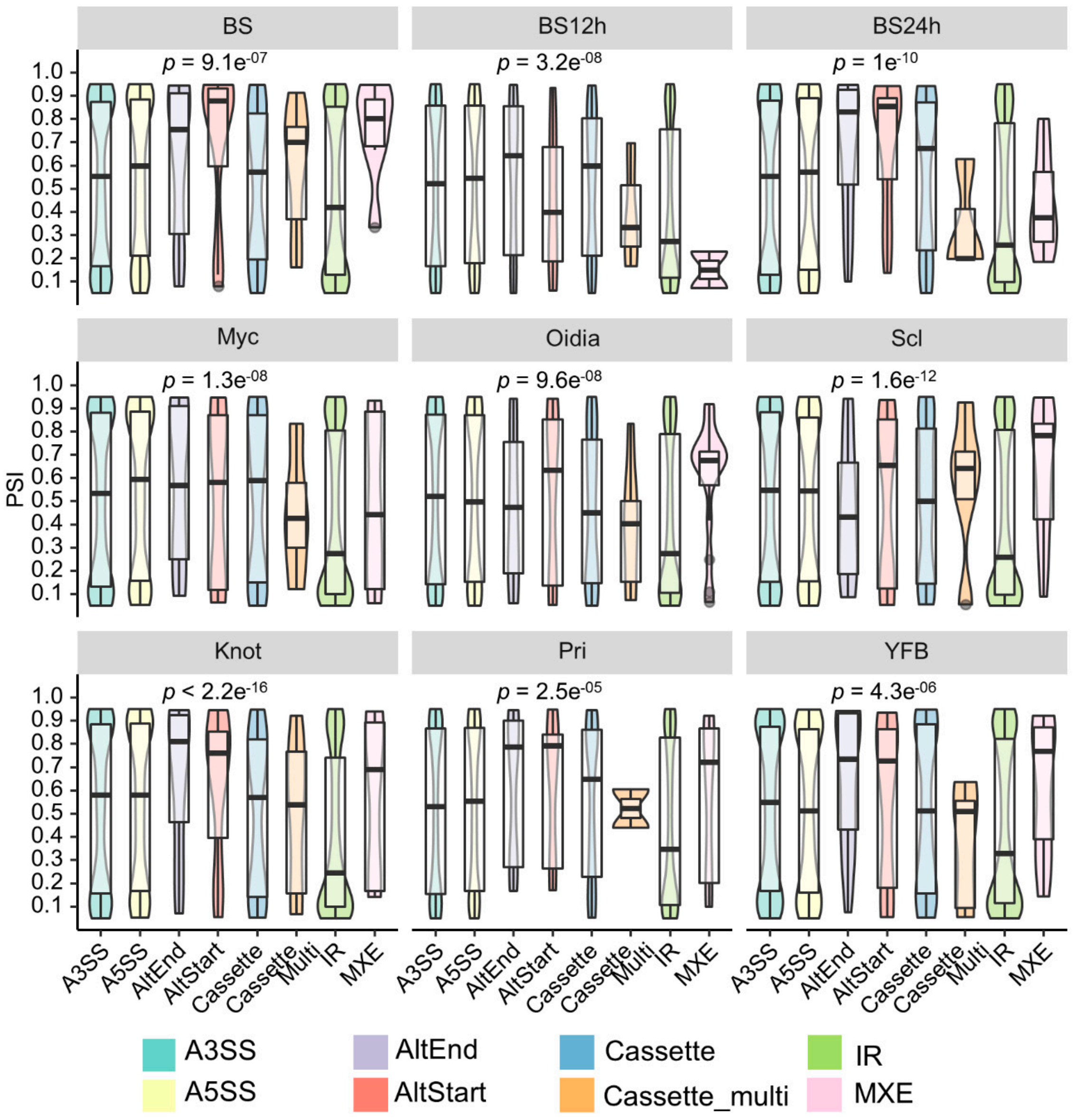

Figure S8. PSI score of eight AS types in nine developmental stages.

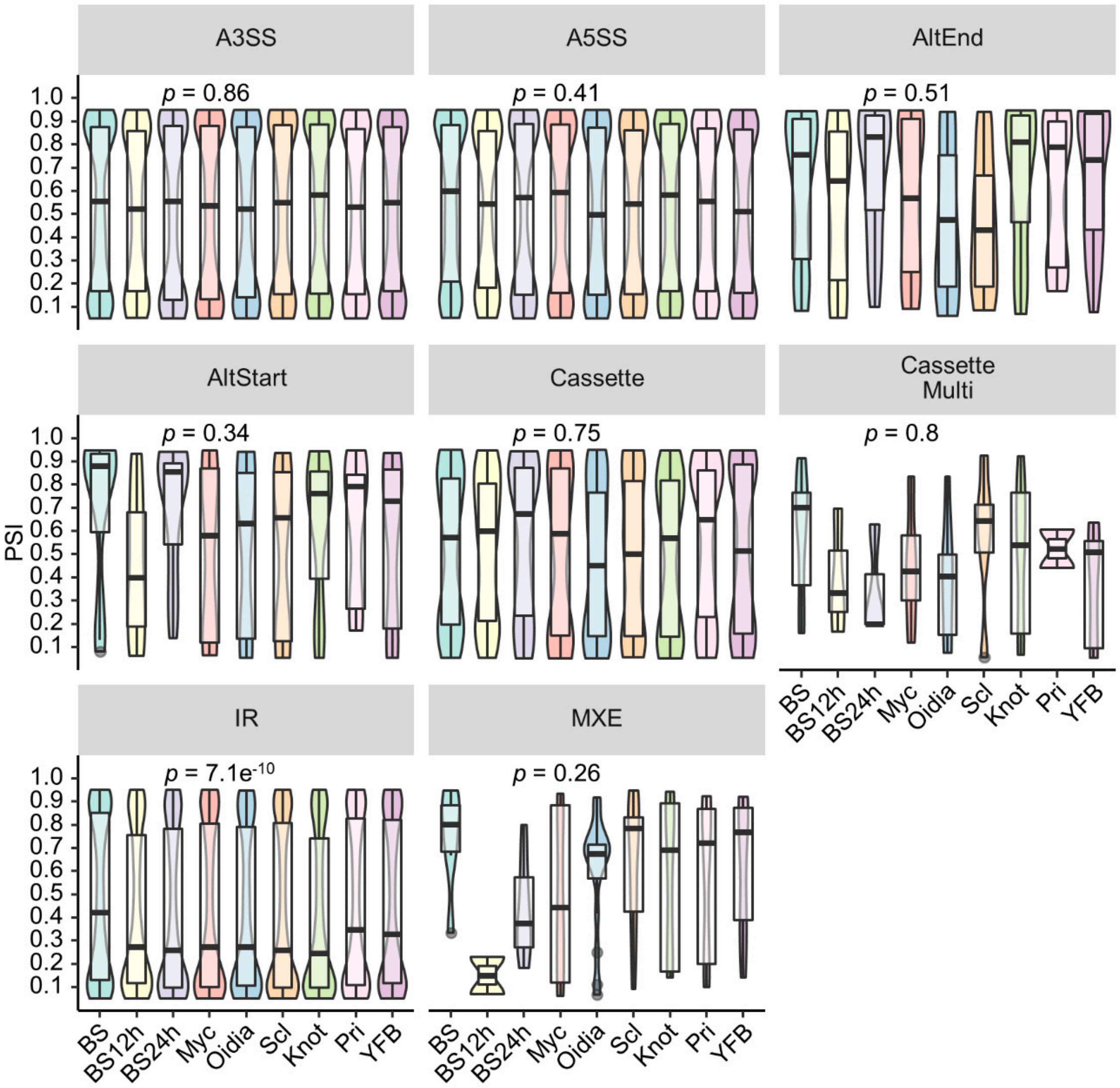

Figure S9. PSI score of nine developmental stages in eight AS types.

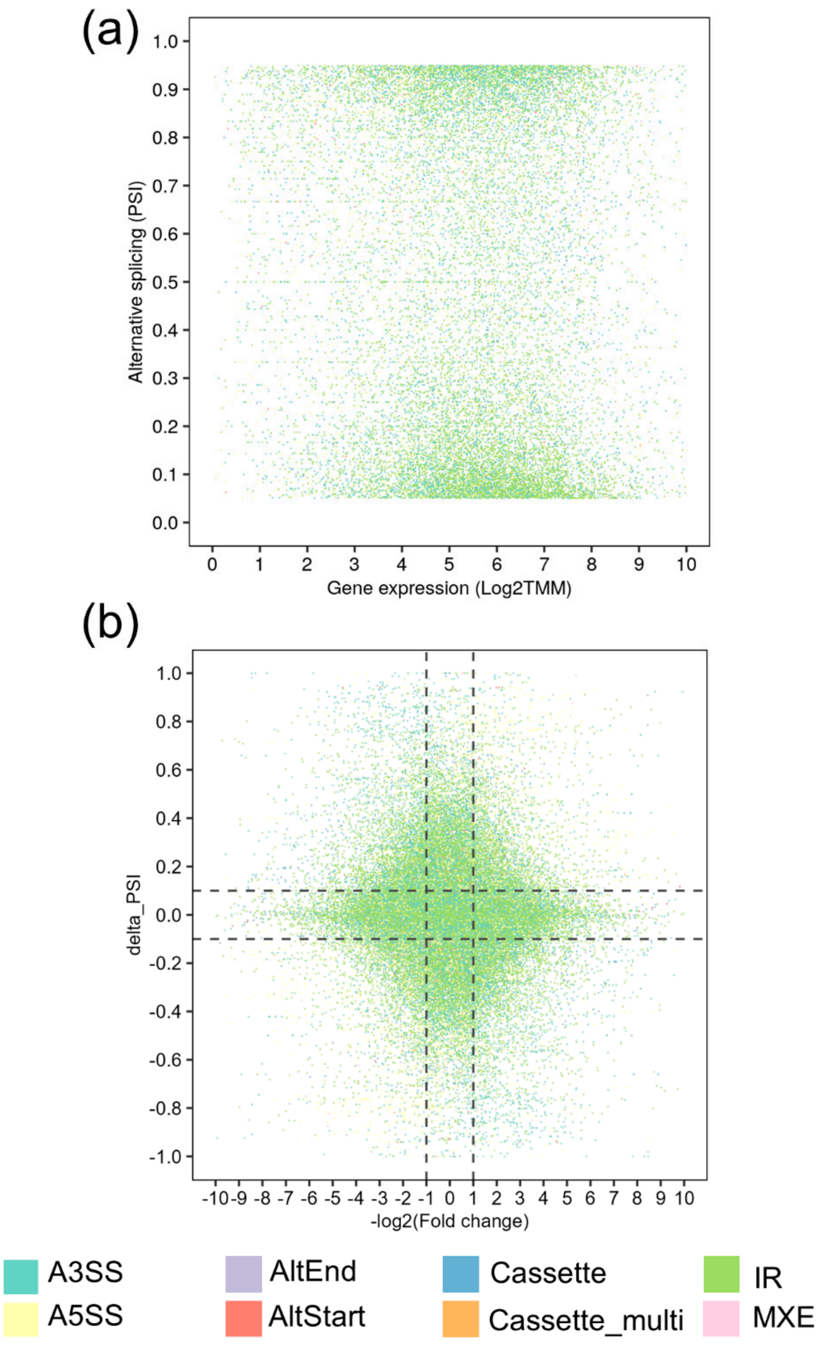

Figure S10. Quantitative relation of gene expression and PSI value of alternatively spliced genes. (a) Scatter plot on gene expression levels and PSI scores; (b) Scatter plot on changes of gene expression levels and PSI scores.

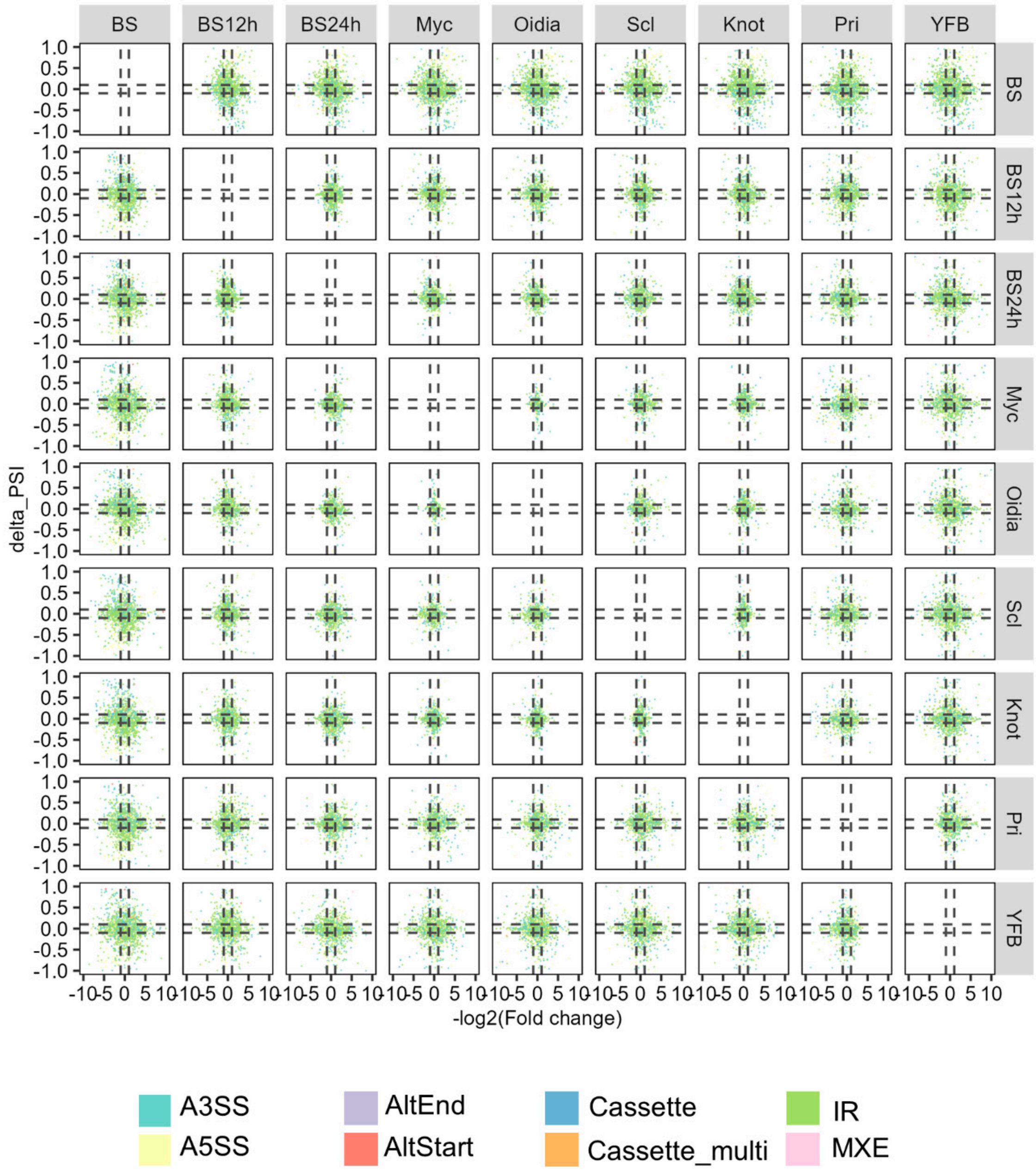

Figure S11. Scatter plots showing the changes on expression levels of alternative spliced genes and their PSI scores. Sample of each column were compared against sample of each row.

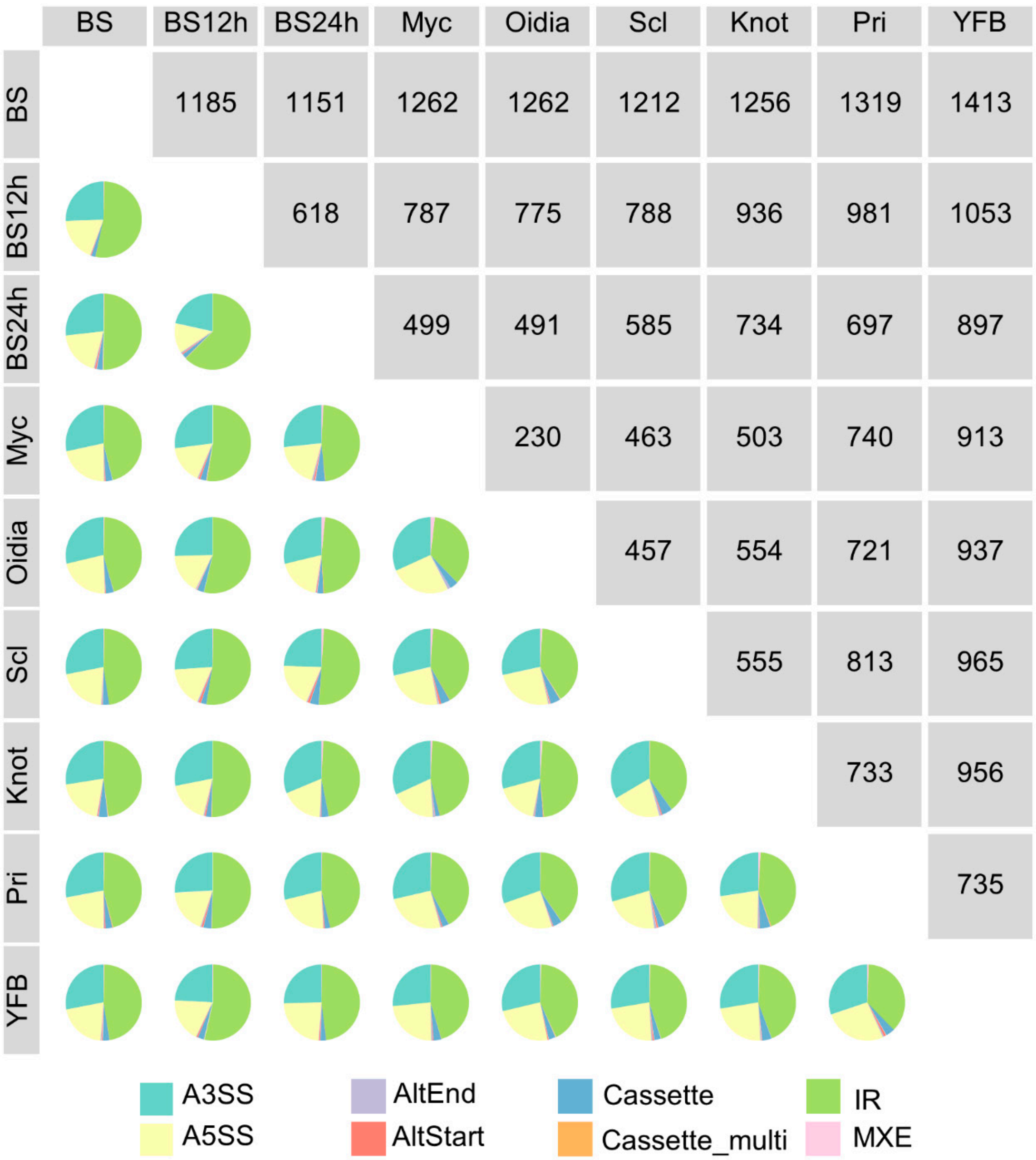

Figure S12. Summary on differentially spliced events between stages. Right upper triangle indicated the number of differentially spliced regions, left lower triangle showed the proportion of AS types.

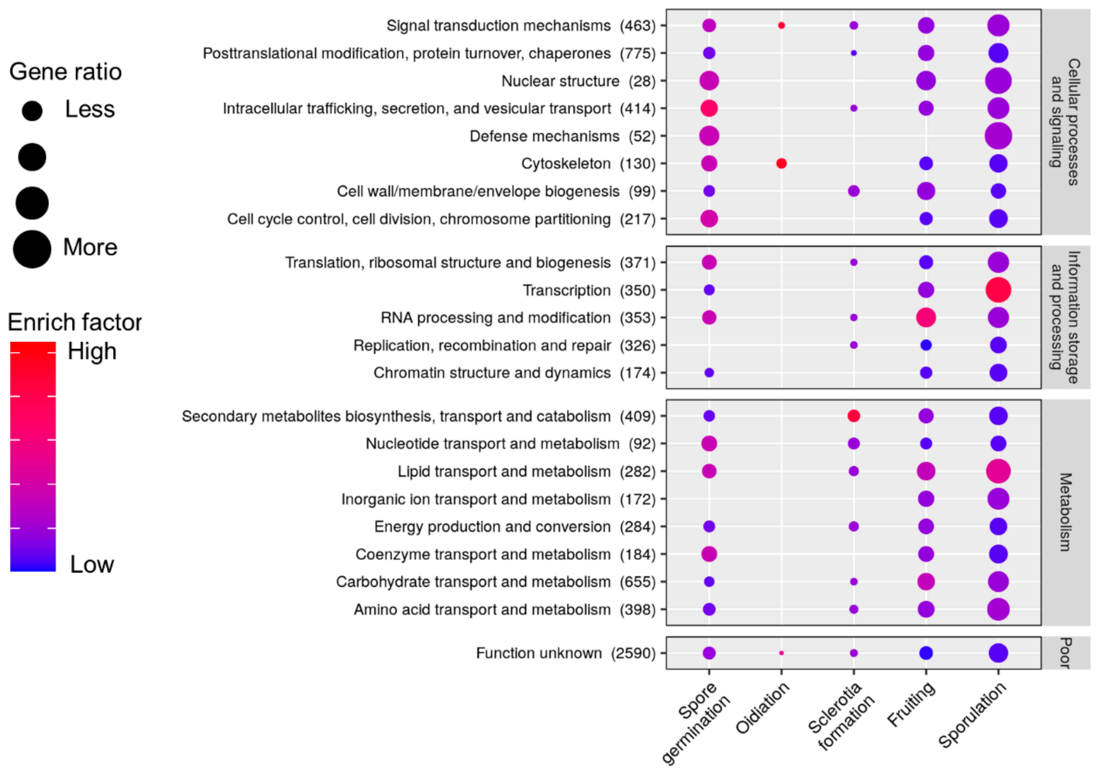

Figure S13. KOG annotation on developmentally regulated alternative splicing specific to the developmental process.

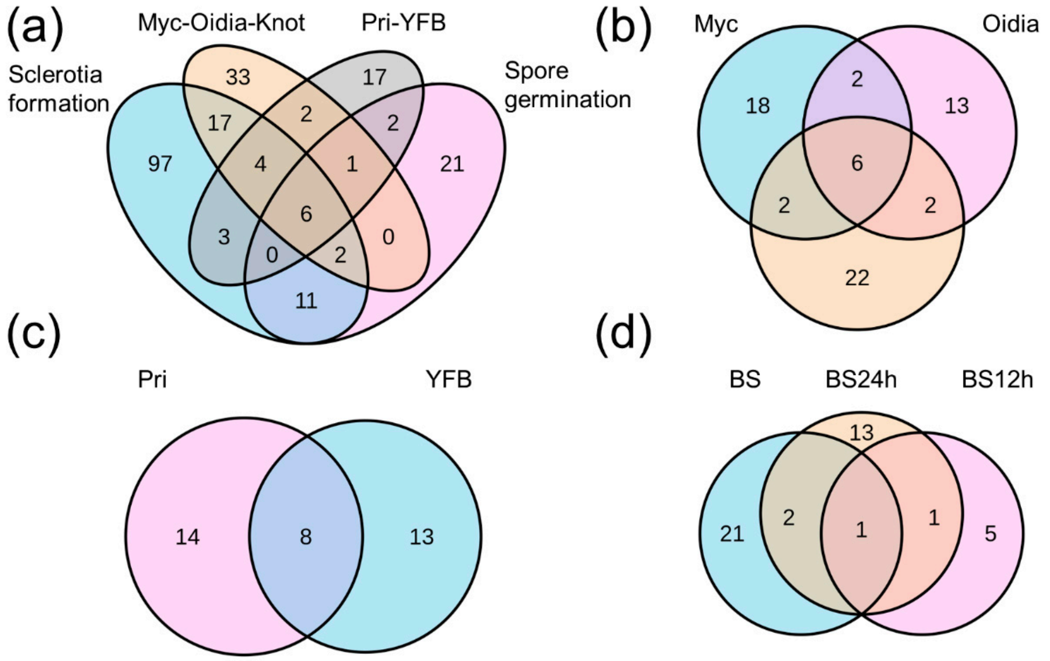

Figure S14. Common RNA editing sites among different stages. (a) Shared RNA editing sites among four major clades; (b) Vegetative mycelia, oidia forming mycelia, mycelia with hyphal knots; (c) Primordia and young fruiting bodies; (d) Spore germination.

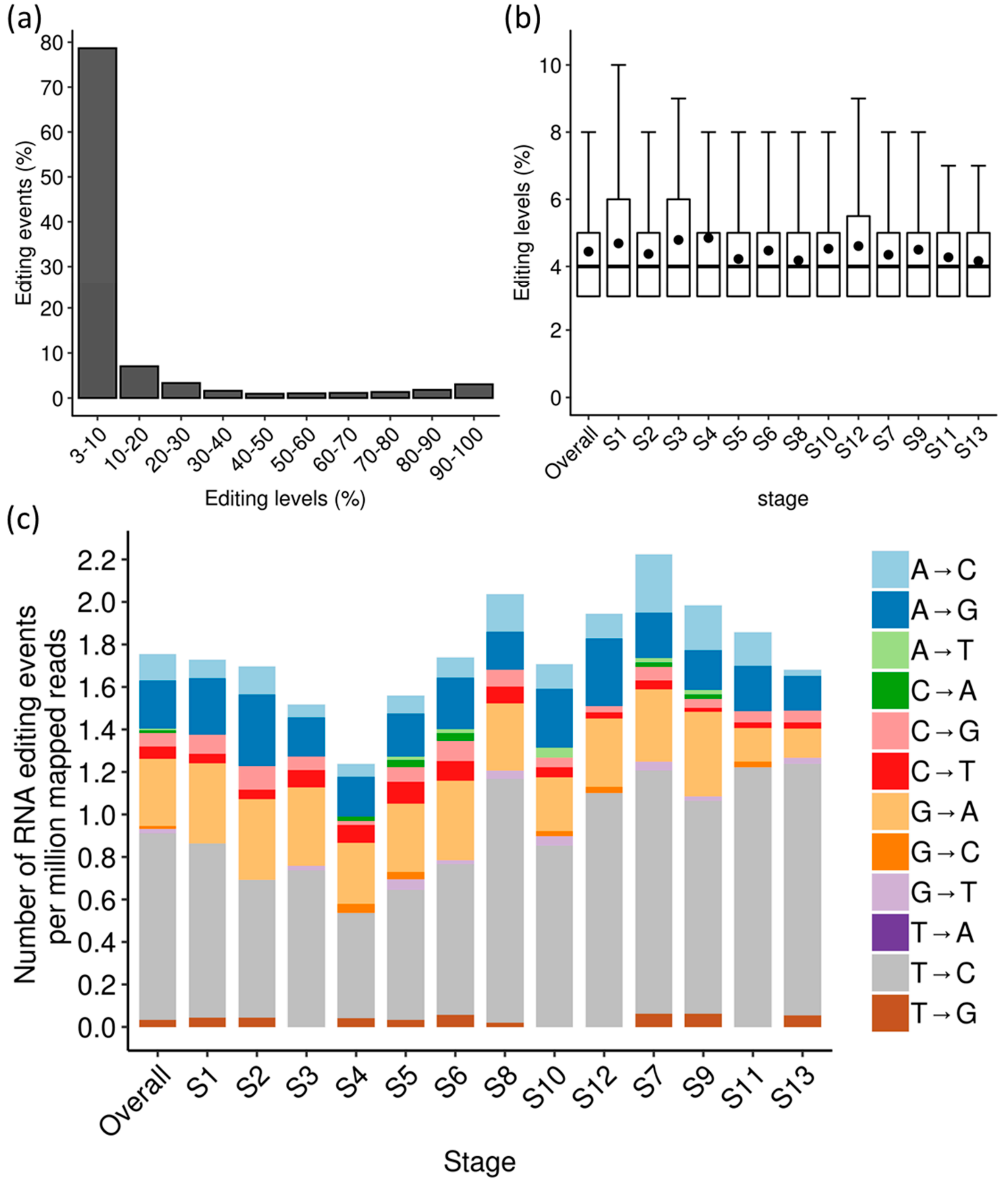

Figure S15. Statistics on RNA editing sites identified from Muraguchi et al. (2015). (a) Histogram showing editing the frequency of 1050 RNA editing events. (b) Box plots showing RNA editing levels of RNA editing events in different stages/tissues. (c) The number of each type of RNA editing events per million mapped reads in different developmental stages. Sequencing data was summarised in Table S10.

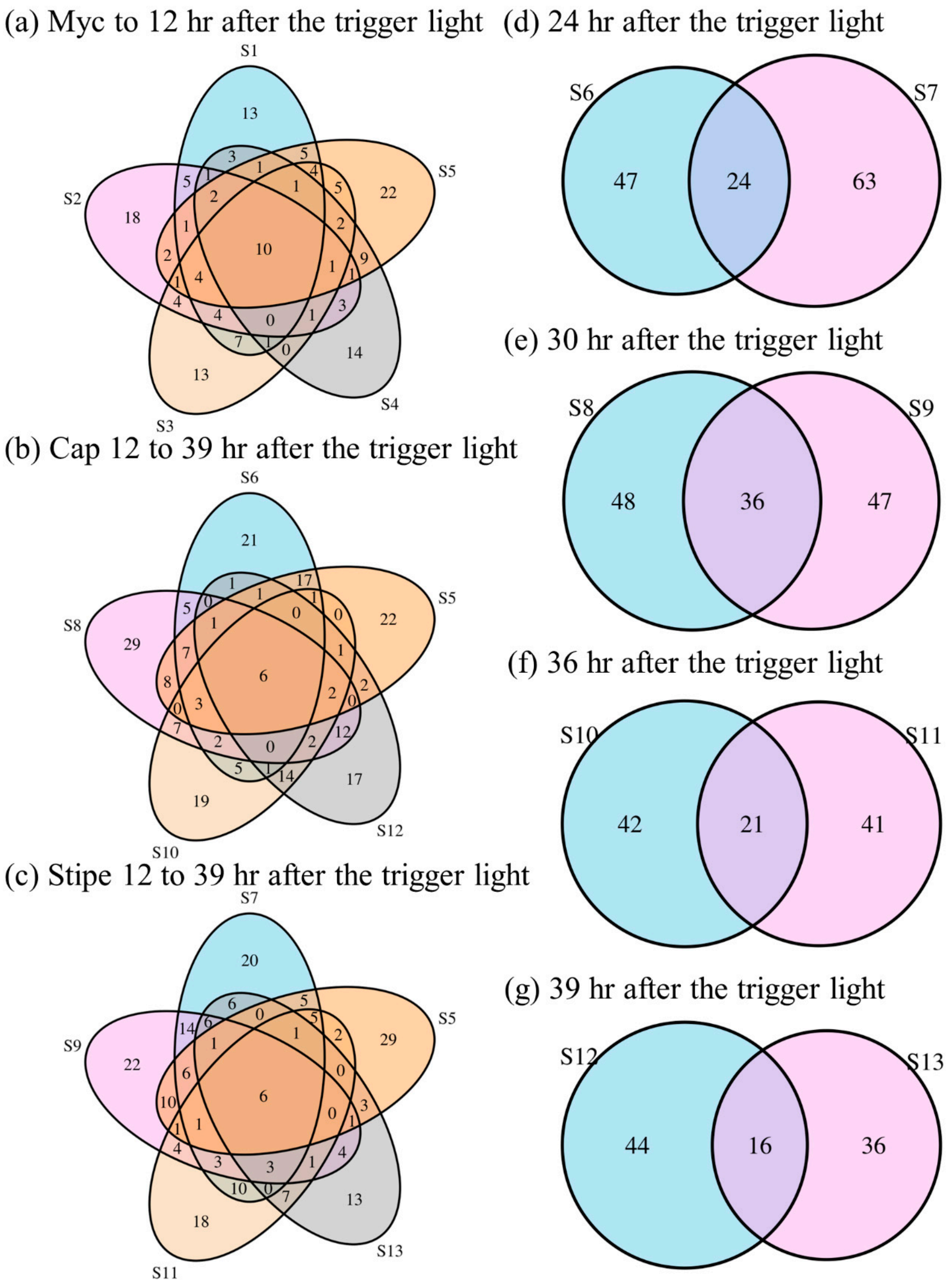

Figure S16. Common RNA editing sites identified from transcriptome data generated by Muraguchi et al. (2015). (a) Vegetative mycelium to fruiting body primordia 12 h; (b) Cap of fruiting body from 12 h to 39 h; (c) Stipe of fruiting body from 12 h to 39 h; (d-g) fruiting body, 24 h/30 h/36 h/39 h after the light triggering.

Sample	Editing level	Reverse	Forward
**DNA**	-		
**M1**	0		
**M2 ***	0.19		
**M3**	0.06		
**K1 ***	0.18		
**K2***	0.04		
**K3 ***	0.7		

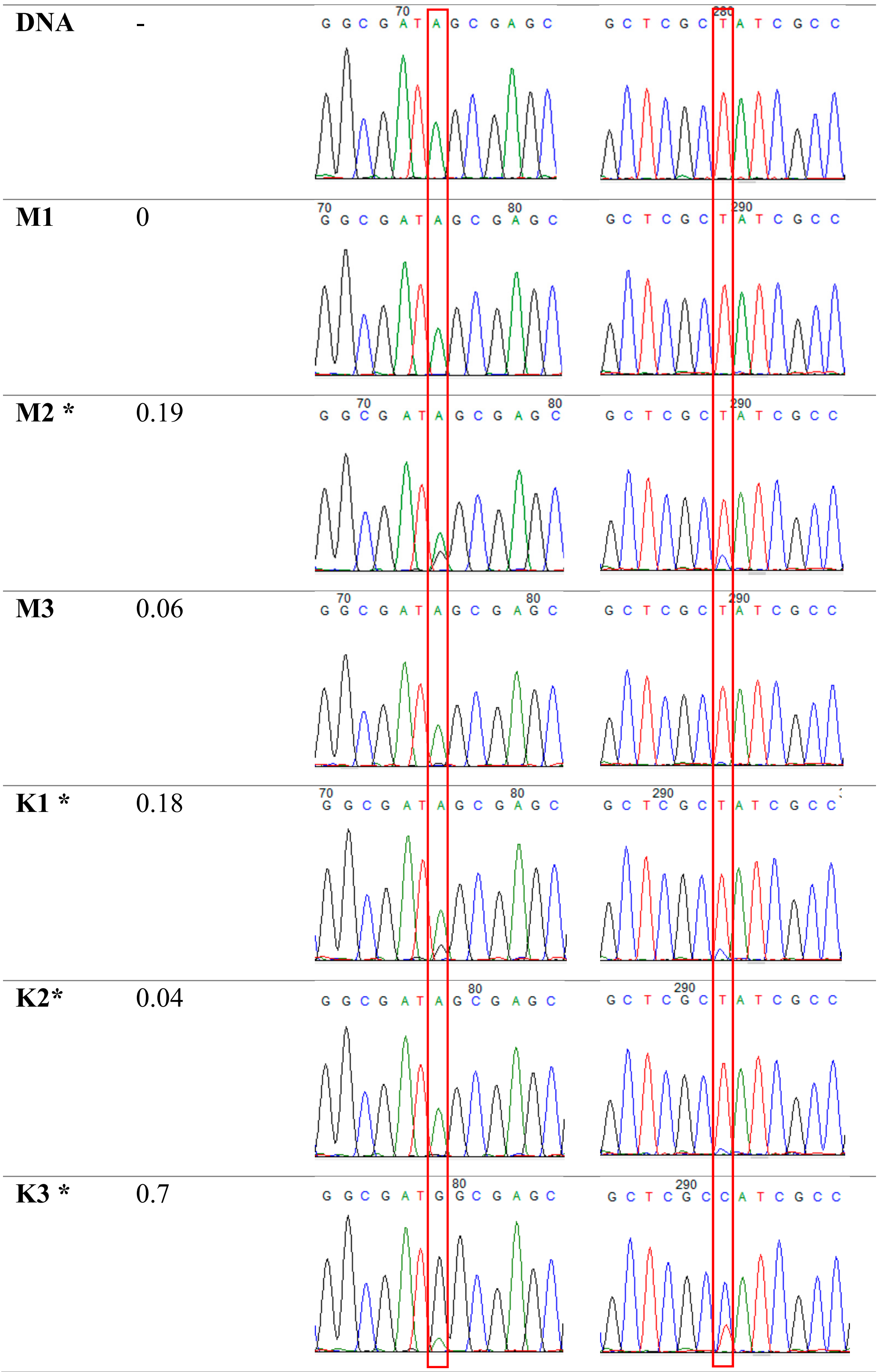

Figure S17. Validation of RNA editing on hypothetical protein (CC2G_003350, scaffold_11: 1716350U>C).

Sample	Editing level	Reverse	Forward
**DNA**	-	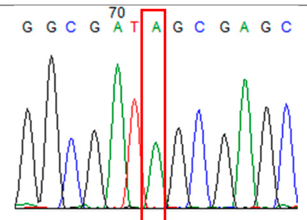	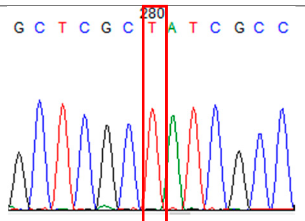
**O1**	0	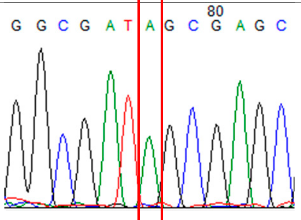	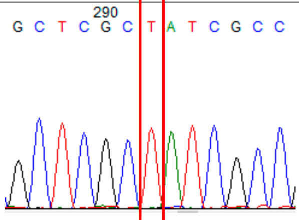
**O2 ***	0.12	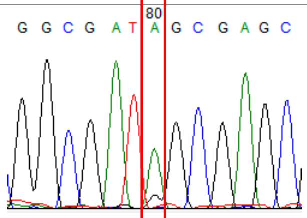	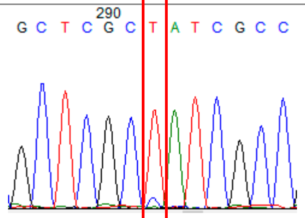
**O3**	0.07	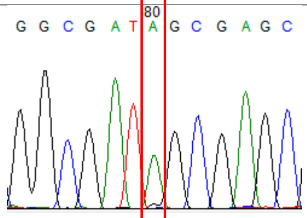	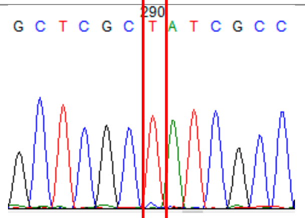
**S1 ***	0.06	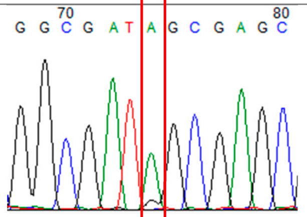	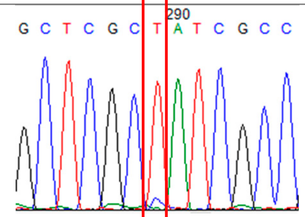
**S2**	0.03	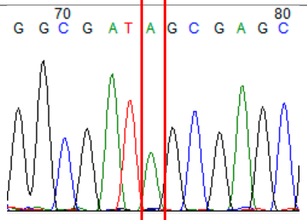	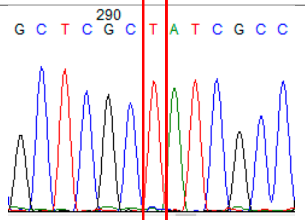
**S3**	0	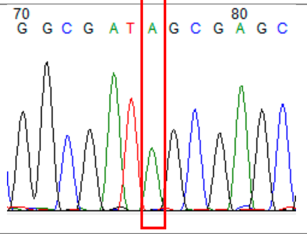	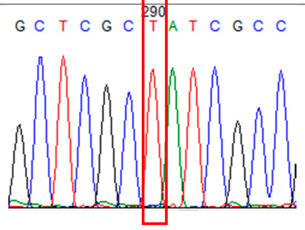

Figure S17. Validation of RNA editing on hypothetical protein (CC2G_003350, scaffold_11: 1716350U>C), continued.

Sample	Editing level	Reverse	Forward
**DNA**	-	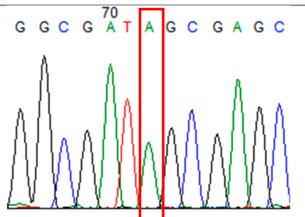	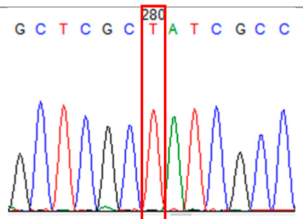
**P1**	0	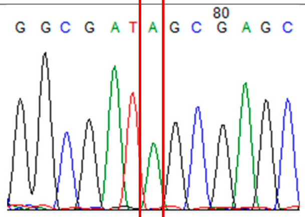	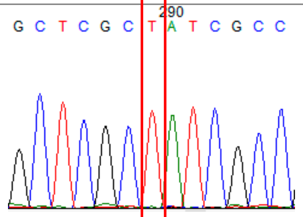
**P2**	0	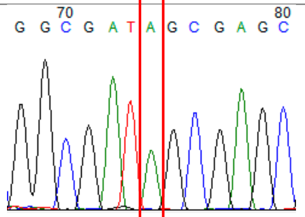	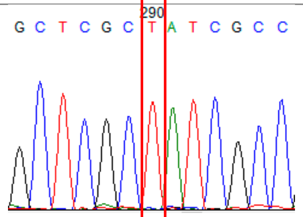
**P3**	0	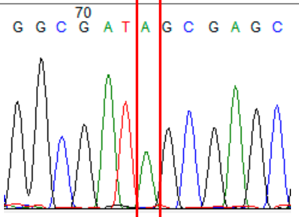	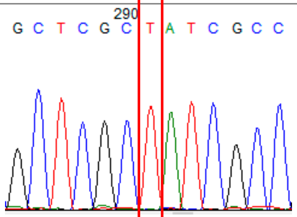
**Y1**	0	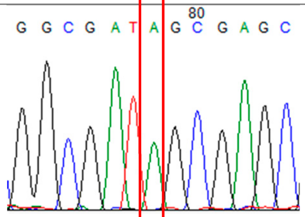	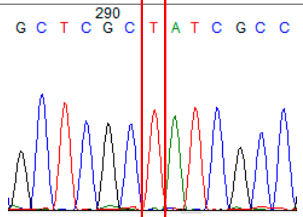
**Y2**	0	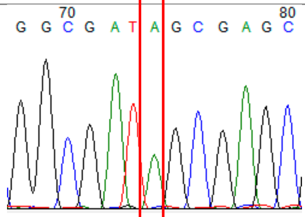	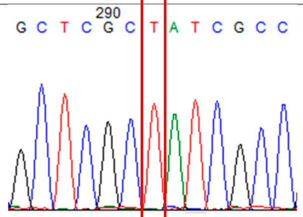
**Y3**	0	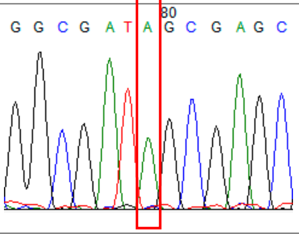	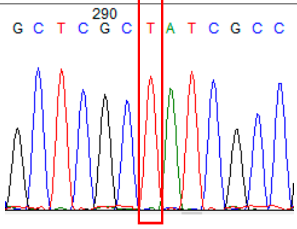

Figure S17. Validation of RNA editing on hypothetical protein (CC2G_003350, scaffold_11: 1716350U>C), continued.

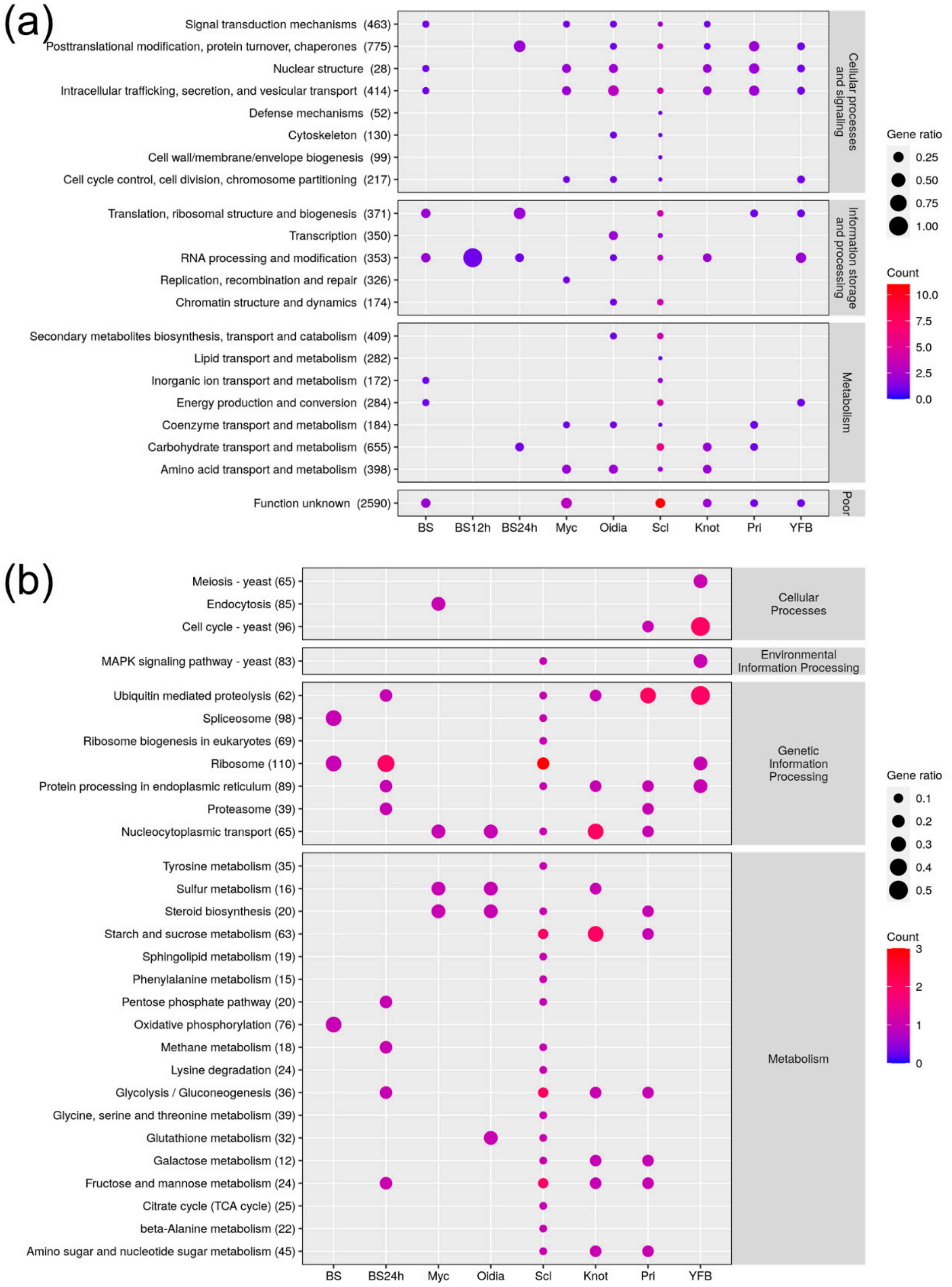

Figure S18. Functional annotation of genes with RNA editing. Gene ratio is calculated by annotated genes of specific functional term in each stage over annotated genes of each stage in the genome background. (a) KOG term annotation; (b) KEGG pathway annotation.

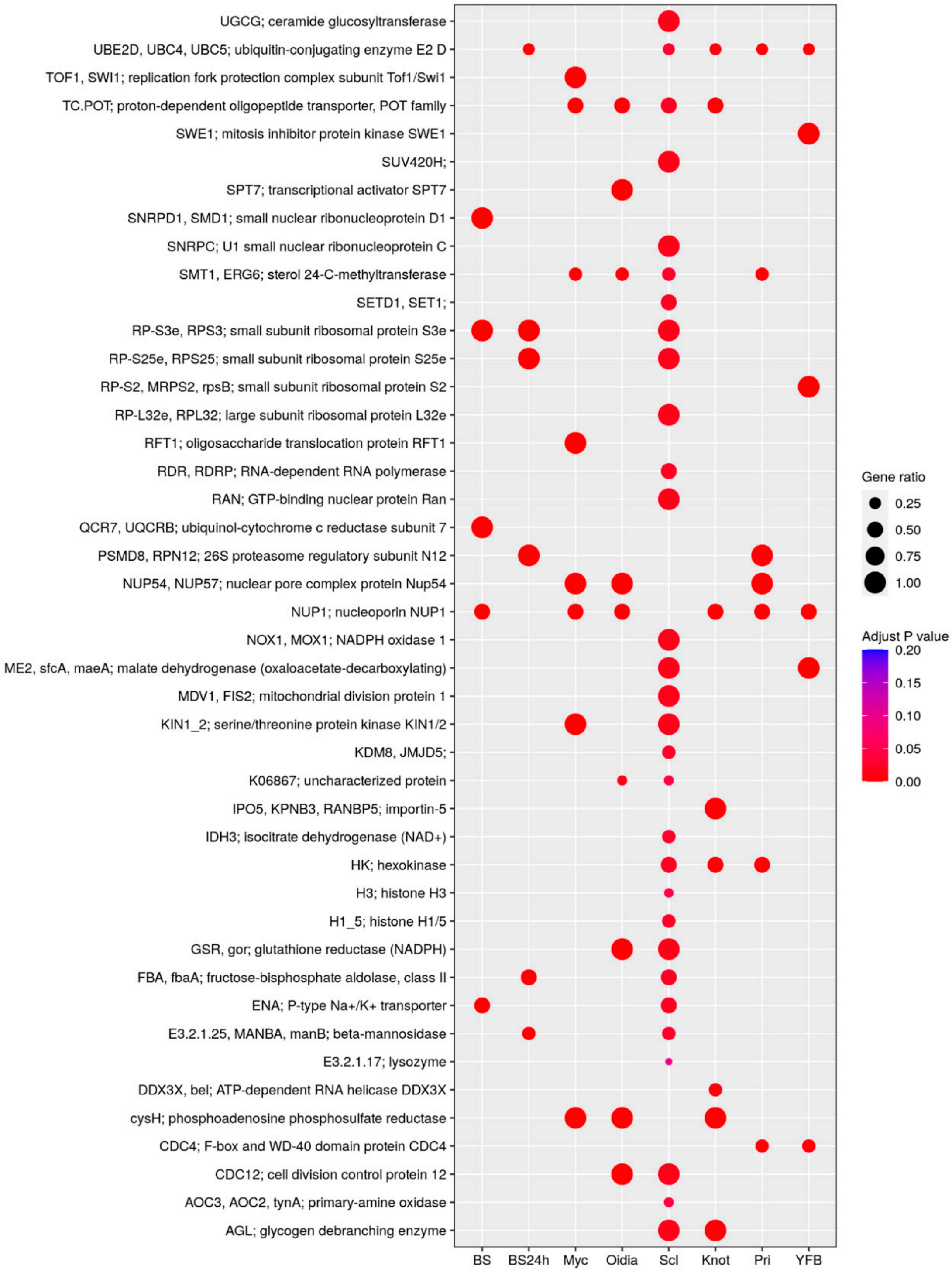

Figure S19. KO annotation of genes with RNA editing. Gene ratio is calculated by annotated genes of specific KO term in each stage over annotated genes of specific KO term in the genome background. Enriched groups with Benjamini and Hochberg method (BH) adjusted p value ≤ 0.20 are coloured red to blue.

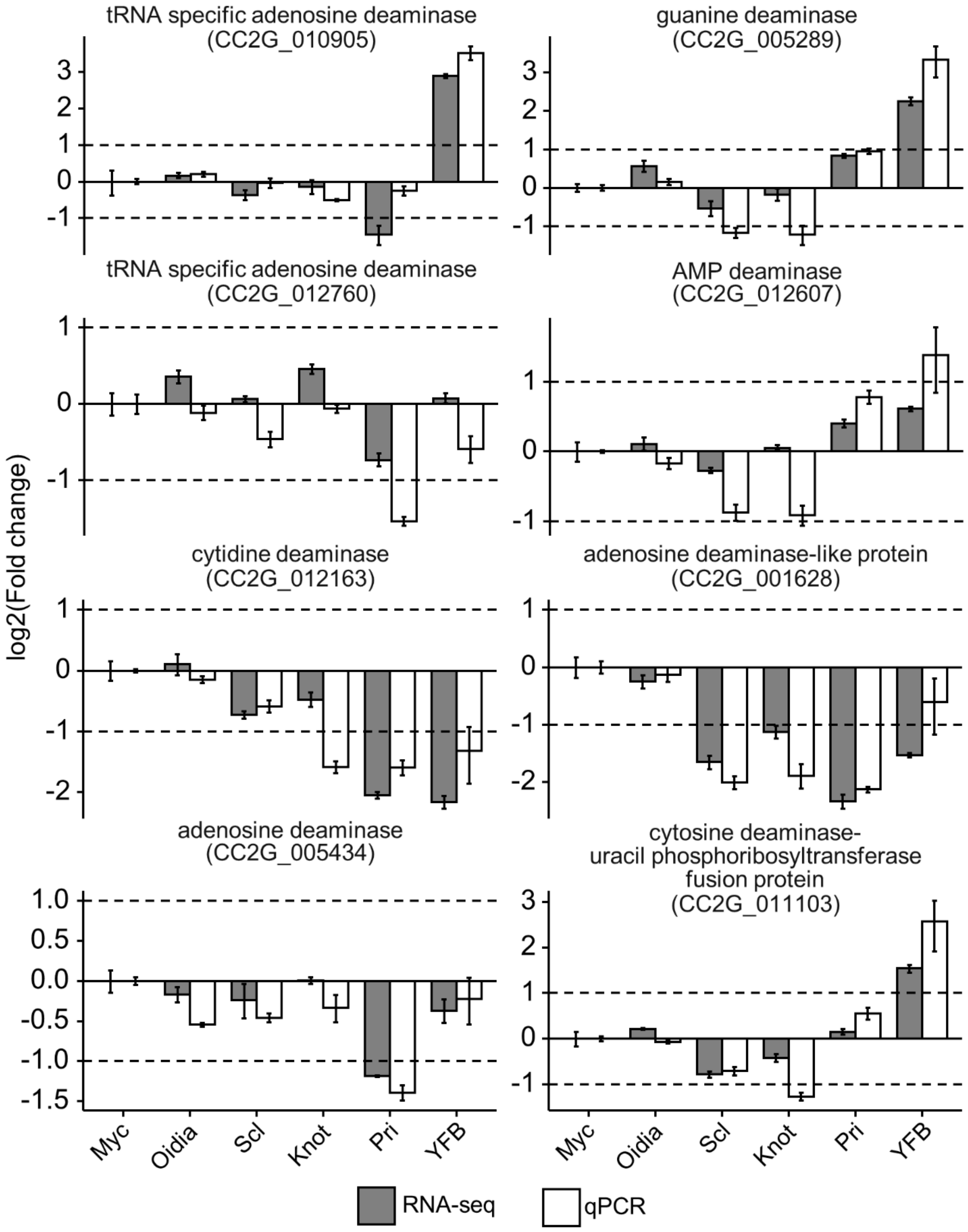

Figure S20. Expression levels of nucleoside deaminase annotated in *C. cinerea*. Expression fold changes are compared to vegetative mycelia (Myc).
